# Validation of a Short-Form Five Facet Mindfulness Questionnaire Instrument in China

**DOI:** 10.3389/fpsyg.2019.03031

**Published:** 2020-01-17

**Authors:** Yong Meng, Kaixian Mao, Chaoping Li

**Affiliations:** ^1^Management Institute, Xinxiang Medical University, Xinxiang, China; ^2^Department of Management, Hong Kong University of Science and Technology, Kowloon, Hong Kong; ^3^Institute of Organization and Human Resources, Renmin University of China, Beijing, China

**Keywords:** mindfulness, five facet mindfulness questionnaire, validation, servant leadership, work-family balance

## Abstract

The Five Facet Mindfulness Questionnaire (FFMQ) developed by Baer and colleagues has been widely used owing to its satisfactory psychometric properties. Because there was not yet a short-form version of the FFMQ (SF-FFMQ) that could be utilized in work settings available in China, we developed a Chinese SF-FFMQ. Exploratory factor analysis (EFA) and confirmatory factor analysis (CFA) supported a five-factor structure of our Chinese SF-FFMQ in two Chinese samples (Sample 1, *N* = 535; Sample 2, *N* = 391). The internal consistencies of the facets and the whole scale were acceptable. The predictive validity of this questionnaire was affirmed. Overall, the mindfulness facets correlated with related constructs including depression symptoms, anxiety symptoms, employee life well-being, employee workplace well-being, and employee psychological well-being. In sample 2, which consisted of participants employed by local companies, we found that mindfulness mediated a positive relationship between servant leadership and employees' satisfaction with work-family balance. In conclusion, our Chinese SF-FFMQ was found to be a valid and reliable measurement tool and our results support its use in both research and practice in clinical and work settings in China.

## Introduction

Mindfulness, which has ancient roots in Buddhism and may be unfamiliar or even a somewhat mysterious concept to many Westerners, refers most simply to the state of concentrating fully on what is happening in the present and being aware of one's current experiences without reactivity or judgment (Brown and Ryan, 2003; Baer et al., 2006; Eby et al., 2019). Mindfulness practices have been shown to help reduce anxiety symptoms, depression symptoms, and sadness, and to be useful for modulating one's motivations and behaviors in a broad sense (Kabat-Zinn, 1982, 1994; Brown and Ryan, 2003; Creswell and Lindsay, 2014; Papies et al., 2015). Owing to these benefits, mindfulness-based interventions have emerged in medical settings and such interventions have yielded positive effects on physical and mental health (Creswell, 2017; Wielgosz et al., 2019). Recently, researchers in various areas of psychology, including social psychologists as well as industrial and organizational psychologists, have shown increasing interest in mindfulness and preliminary findings suggesting that it can play an active role in improving interpersonal and work settings (Good et al., 2016). Notably, there has been a growth in interest in the possibility that management science could develop mindfulness training to enable managers to improve organizations (Dane, 2011; Glomb et al., 2011; Good et al., 2016; Yu and Zellmer-Bruhn, 2018).

Mindfulness can be conceptualized as a trait or a state, resulting in two types of measurements. The Mindful Attention Awareness Scale (MAAS) was developed to assess mindfulness as a dispositional trait, though its authors also provide an adapted version to measure state mindfulness (Brown and Ryan, 2003). Meanwhile, the Toronto Mindfulness Scale (Lau et al., 2006) is a favored instrument for measuring one's capacity to invoke a mindfulness state. The appropriateness of such instruments, however, depends on how we conceptualize and operationalize mindfulness, including what elements should be considered integral to mindfulness (Quaglia et al., 2015). Some have argued that “acceptance” and “non-judgment” are not fundamental parts of mindfulness (Bodhi, 2011; Dreyfus, 2011), but rather as benefits of mindfulness. In this view, only enhanced attention and awareness are critical for the experience of mindfulness; the fundamental nature of these elements has been reflected in developed scales (e.g., Brown and Ryan, 2004). Notwithstanding, the most appropriate mindfulness scale may depend on the particularly characteristics of the research, including the study population, cohort starting points, and intended applications (Quaglia et al., 2015). The inclusion of derivative elements, such as “non-judgment,” in a measurement tool may be helpful for practical applications (Kabat-Zinn, 2011).

The Five Facet Mindfulness Questionnaire (FFMQ), a multifactorial scale developed by Baer et al. (2006), has been widely used owing to its practical psychometric properties. The five factors of the FFMQ are: *observe, describe, act with awareness, non-judging of inner experience*, and *non-reactivity to inner experience*. In this conceptualization, the term observe refers to one's ability to notice and attend to his or her perceptions, feelings, and thoughts. The term *describe* refers to one's ability to label his or her feelings, sensations, and experiences with words. *Act with awareness* refers to being attentive to activities and being able to avoid distraction. *Non-judging of inner experience* (*non-judging* from her forward for simplicity) means that one does not make judgments about his or her experiences, feelings, and thoughts. Finally, the term non-reactivity to inner experience (*non-reactivity* from here forward for simplicity) refers to one's ability to perceive and notice his or her own feelings, emotions, and thoughts without reacting to them. The original FFMQ has been shown to have good internal consistency and construct validity; and the positive and negative correlations of FFMQ mindfulness facets with related constructs indicate that this scale can be used to predict psychological symptoms (Baer et al., 2006). Although there have been some unexpected findings related to the *observe* facet, the conceptualization of mindfulness employed in the FFMQ is promising for practical applications (Baer et al., 2006, 2008).

An abbreviated version of the FFMQ is warranted because the time burden of the original 39-item version limits its application. In a recent study, Baer et al. (2012) demonstrated adequate internal consistency of a short-form version of FFMQ (SF-FFMQ) with only three items per facet reduced the burden placed upon participants. Utilizing this SF-FFMQ, the authors found that weekly improvement in mindfulness skills mediated a positive relationship between mindfulness-based stress reduction and perceived stress. The psychometric properties of the SF-FFMQ, however, need to be further confirmed.

Dane and Brummel (2014) found that workplace mindfulness related positively to job performance and negatively to turnover intention. They further showed that mindfulness can contribute to job performance even after accounting for the work engagement dimensions. These results indicated that if employees situated their mindsets in the present in the workplaces, they could be more concentrative and in turn achieve better work outcomes. Hülsheger et al. (2013) demonstrated that mindfulness, acting as a mediator, helped reduce employees' emotional exhaustion and increased their job satisfaction. Hülsheger et al. (2014) further showed that mindfulness at work can predict employees' sleep quality and this dynamic relationship was mediated by psychological detachment. Mindfulness in the workplace may also contribute to leaders' psychological capital and, in turn, enact benefits upon their mental health (Roche et al., 2014). Furthermore, mindfulness was reported to mitigate the effects of injustice on rumination and negative emotions and to reduce employees' retaliatory behaviors in the workplace (Long and Christian, 2015) and reduce their work-family conflict (Kiburz et al., 2017). However, few studies have linked leadership theories with mindfulness, leaving an important gap to be filled. Notably, Eisenbeiss and van Knippenberg (2015) found that mindfulness moderated the effects of ethical leadership on the expenditure of extra effort and helpful behaviors, such that employees with higher levels of mindfulness were more responsive to ethical leadership and behaved more ethically.

The present study had two purposes. First, we sought to validate the psychometric properties and facet structure of the first Chinese version, to our knowledge, of the SF-FFMQ. In this context, we aimed to examine the incremental validity of the *observe* facet in light of the unexpected relationships that it showed with some psychological symptoms in Baer et al.'s (2006) study. Second, with the long-term goal of integrating mindfulness training in workplaces, we investigated the utility of the SF-FFMQ in management science and industrial psychology. Baer et al. (2006; 2012) prior studies enrolled student samples, leaving a need for SF-FFMQ in broader populations. Doing so may enable empirical application of mindfulness in leadership theories and shape our understanding of mindfulness as a potential root construct in management studies (i.e., a construct with fundamental impacts on people's emotions, thoughts, and behaviors; Good et al., 2016).

## Overview of the Current Study

We first validated our Chinese SF-FFMQ and examined its factorial structure. The internal consistency of the scale and each of its five facets were examined, and we explored correlations of the five mindfulness facets with psychological measures. There has been a recent increase in interest in using mindfulness-based interventions—such as Mindfulness-Based Stress Reduction Programs (Kabat-Zinn, 1990) and Mindfulness-based Cognitive Therapy (Segal et al., 2002)—which have been shown to relate positively to psychological states, including anxiety, stress, and depression. Building upon prior studies that explored the relationship between mindfulness and psychological symptoms (Jermann et al., 2009; Gu et al., 2016), we explored links between SF-FFMQ scores and standard psychological symptomology assessment scores.

In addition, we investigated the role of mindfulness skills in the relationship between the leadership theory known as servant leadership and employee well-being. Based on “the natural feeling that one wants to serve, to serve first” (Greenleaf, 1977, p. 7), servant leadership refers to servant leaders' desire to serve and lead at the same time (Sendjaya and Sarros, 2002). Servant leadership places the best interests of others over the interests of leaders (Laub, 1999, p. 81). It differs from other leadership theories (e.g., transformational leadership, authentic leadership, and ethical leadership) in that it stresses the importance of serving followers, caring for their needs, and helping them grow (van Dierendonck, 2011; Sousa and van Dierendonck, 2017). Servant leadership encompasses listening and understanding followers and showing empathy and recuperation for employees (Spears, 1995, 2004; Laub, 1999). We are interested in examining whether mindfulness skills may enhance servant leadership adherents' propensity to benefit from their servant leadership behaviors. Employees may undergo personal growth that leads to more salubrious outcomes both in the workplace and in their personal lives. The results of this line of work may help to elucidate the role of mindfulness in management research, especially in leadership studies, and advance our understanding of servant leadership theory.

Specifically, we argue that servant leadership can contribute to followers' mindfulness by facilitating their self-acceptance and helping them grow professionally and spiritually (Spears, 2004), which can further lead to their work and family balance. First, the climate and culture that servant leadership behaviors create are useful to nurture subordinates (Liden et al., 2014), which can significantly fuel employee psychological well-being (van Dierendonck et al., 2014). Since servant leadership behaviors use listening, empathy, and healing to interact with followers (Spears, 2004), followers can gradually nurture the habit of mindfulness and behave in a mindful way.

Further, since servant leaders display authenticity and share power with subordinates in workplaces (Laub, 1999) and they develop people by serving needs and nurturing the growth (Spears, 2004; Liden et al., 2014), it becomes natural for followers to fully experience the current. Such characteristics of conceptualization that servant leaders have (van Dierendonck et al., 2014) could even provide directions that help employees to better master the skills of concentration and experiencing without reactivity or judgment. In turn, such mindfulness skills could be beneficial when employees deal with the work-family issues such as conflicts (Greenhaus et al., 2003). If employees can adopt the way of fully experiencing without judgment, they can better allocate their time and even achieve better work-family enrichment (Vieira et al., 2018). As a result, these employees can feel more satisfied with their life quality and work-family balance (Lyu et al., 2019). Thus, we would expect that mindfulness mediates the positive relation between servant leadership and employee's satisfaction with work-family balance.

## Methods

### Participants

We enrolled two samples in the current study. We involved a total of 535 participants in Sample 1. All participants were recruited via an online forum in the largest online business and management community for people in China, JG.COM (https://bbs.pinggu.org). We posted study announcements inviting community members to complete our surveys for 50 “gold coins” that serve as currency in the online forum. All respondents were reminded that they should respond to all questions carefully and that researchers would check the surveys for completeness, which was a requisite for the 50 gold coin compensation. Of 581 submitted surveys, 535 were confirmed to be valid. These 535 participants (226 males and 309 females) had a mean age of 23.53 years [standard deviation (SD) = 5.28; range, 18–60].

Sample 2 consisted of manufacturing industry employees in Henan province in central China. We reached out to upper-management team members of target companies. With their approval, we distributed our questionnaires at staff meetings. All participants were informed of the purpose of this study and that their participation was anonymous and confidential, and given instructions for how to fill out the questionnaire. The study's anonymity and confidentiality were reaffirmed on the cover page of the questionnaires, which were collected immediately upon completion.

Of 423 distributed questionnaires, we received 415 filled-out questionnaires back (response rate = 98.11%), indicating that the participation incentive was effective. The final sample consists of 391 participants because 24 respondents returned questionnaire with some invalid or incomplete responses. Of the 391 participants in the final sample 2 cohort, 314 were male (80.3%) and 77 were female (19.7%). They had a mean age of 38.35 years (SD = 8.86) with a mean tenure of 17.16 years (SD = 9.89). Their educational backgrounds were as follows: associate's degree or less, *N* = 279 (71.4%); bachelor's degree, *N* = 99 (25.3%); and graduate degree, *N* = 13 (3.3%). Their employing organizations were as follows: state-owned enterprise, *N* = 44 (11.3%); private enterprise, *N* = 146 (37.3%); and foreign-funded enterprise or Sino-foreign joint venture, *N* = 201 (51.4%).

### Materials

We followed Brislin's (1980) translation and back-translation procedure to obtain Chinese versions of all originally-in-English instruments used in this study. First, a bilingual PhD student translated the scale items into Mandarin. Then, another bilingual Ph.D. student back-translated the Mandarin items back into English. All three authors compared the two sets of English items to detect any discrepancies or inconsistencies, which were discussed and corrected with minor adjustments.

#### SF-FFMQ

The original FFMQ has five factors and consists of 39 items, which were based on a composite of items from previous mindfulness scales, such as the MAAS, Freiburg Mindfulness Inventory, and Kentucky Inventory of Mindfulness Skills subjected to exploratory factor analysis (EFA) and confirmatory factor analysis (CFA). Each factor has eight items, except *non-reactivity*, which has seven items. Here, we used a 20-item SF-FFMQ with four items per factor, chosen based on factor loadings and specific contents (Baer et al., 2006). Participants were asked to rate whether each item reflected the facts of their work and lives on a 5-point Likert scale ranging from 1 (never) to 5 (always).

#### Depression

We assessed depression symptoms with the Center for Epidemiological Studies Depression Scale (CES-D; Radloff, 1977), a widely used tool for screening depressive tendencies. This scale was translated and validated in China by Zhang et al. (1987). It has 20 items that reflect a range of symptoms such as loneliness, fear, and sadness. Participants were asked to rate each item on a 4-point Likert scale from 1 (never or a little) to 4 (almost all or all the time). Higher total scores indicate more severe depression symptoms. Reverse code items from the original scale were altered to avoid potential misunderstanding. The CES-D had excellent reliability in this study (Cronbach's alpha = 0.96).

#### Anxiety

The 20-item Self-rating Anxiety Scale (SAS) developed by Zung (1971) was used to evaluate anxiety. The SAS reflects the subjective experience of anxiety. We utilized the Chinese version of SAS published by Zhang (1998). Participants rate the SAS items on a 4-point Likert scale from 1 (never or a little) to 4 (almost all or all the time), with higher total scores indicating more severe anxiety symptoms. Reverse code items from the original scale were altered to avoid potential misunderstanding. The SAS had excellent reliability in this study (Cronbach's alpha = 0.94).

#### Employee Well-Being

We measured employee well-being with an 18-item well-being scale from Zheng et al. (2015) composed of three 6-item well-being domain subscales: life, workplace, and psychological. Participants responded to the scale on a 7-point Likert scale from 1 (strongly disagree) to 7 (strongly agree). The Cronbach's alpha values for the whole scale and the subscales for this study were 0.92, 0.85, 0.90, and 0.86, respectively.

#### Servant Leadership

We administered a 15-item, five-factors Chinese-version servant leadership scale (SLS) based on Barbuto and Wheeler's (2006) original version; the Chinese version was validated by Sun and Wang (2010). Participants responded to each item on a 5-point Likert scale from 1 (strongly disagree) to 5 (strongly agree). We obtained excellent reliability for the whole SLS (Cronbach's alpha = 0.95), with the following factor Cronbach's alpha values: emotional healing, 0.87; persuasive mapping, 0.85; altruistic calling, 0.88; wisdom, 0.85; and organizational stewardship, 0.77.

#### Satisfaction With Work-Family Balance

Satisfaction with work-family balance was assessed using a five-item scale from Valcour (2007) translated to Chinese according to Brislin's (1980) procedure. Participants responded on a 5-point Likert scale from 1 (strongly disagree) to 5 (strongly agree). The scale had good reliability in this study (Cronbach's alpha = 0.86).

### Data-Analysis Strategy

Cronbach's alpha coefficients were calculated in IBM SPSS 24.0. EFA and CFA performed to assess factor structure were completed in IBM Amos 23.0. EFA was conducted with principal axis factoring, an oblique rotation (promax), and the following model-fit indices: the ratio of minimum fit function chi-square to degree of freedom (χ^2^/*df*), goodness of fit index (GFI), normed fit index (NFI), incremental fit index (IFI), Tucker-Lewis index (TLI), comparative fit index (CFI), and root mean square error of approximation (RMSEA). The structural equation modeling method was used to examine the potential mediating role of mindfulness in the relationships of servant leadership and satisfaction with work-family balance, whilst controlling for participant gender, age, educational background, employing organization type, and work tenure.

## Results

### Psychometric Properties and Factorial Structure of the Chinese SF-FFMQ

In Sample 1, EFA with an oblique rotation identified common factors with eigenvalues > 1 were identified, leading us to discern a five-factor structure of the 20 items, with four items per factor. The five factors explained 57.47% of the total variance. The loadings of the items ranged from 0.57 to 0.82 (Table 1).

**Table 1:** EFA item loadings and reliabilities of the five mindfulness facet factors act with awareness (factor 1), describe (factor 2), observe (factor 3), non-judging (factor 4), and non-reactivity (factor 5) (*N* = 535).

**Item**	**Factor**				
	**1**	**2**	**3**	**4**	**5**
FMI 25: I watch my feelings without getting lost in them. 我正视自己的感受, 却不会沉迷其中。				0.62	
MQ 1: Usually when I have distressing thoughts or images, I am able just to notice them without reacting. 通常当我脑海中有痛苦的想法或画面时, 我能够马上察觉到, 但不会立即回应。				0.61	
MQ 9: Usually when I have distressing thoughts or images, I “step back” and am aware of the thought or image without getting taken over by it. 通常当我脑海中有让我痛苦的想法或画面时, 我会“退一步”, 但又不被它们所控制。				0.79	
MQ 10: Usually when I have distressing thoughts or images, I just notice them and let them go. 通常当我脑海中有痛苦的想法或画面时, 我能刚好察觉到它们并坦然放开。				0.70	
KIMS 9: When I'm walking, I deliberately notice the sensations of my body moving. 当我在走路的时候, 有意地去注意身体移动的感觉。			0.74		
KIMS 13: When I take a shower or a bath, I stay alert to the sensations of water on my body. 当我在洗澡的时候, 我时刻留意着水在我身上流动的感觉。			0.76		
KIMS 17: I notice how foods and drinks affect my thoughts, bodily sensations, and emotions. 我注意到食物和饮料如何影。			0.69		
KIMS 21: I pay attention to sensations, such as the wind in my hair or sun on my face. 我注意各种感觉, 例如风拂过头发或阳光洒在脸上的感觉。			0.77		
MAAS 7: It seems I am “running on automatic” without much awareness of what I'm doing. 在“自动运行” 一样, 对自己正在做什么没有清晰的认识。	0.79				
MAAS 10: I do jobs or tasks automatically, without being aware of what I'm doing. 我机械地完成工作任务, 对自己正在做什么缺乏清晰认识。	0.82				
MAAS 14: I find myself doing things without paying attention. 我发现自己做事情的时候并不专心。	0.75				
KIMS 23: I don't pay attention to what I'm doing because I'm daydreaming, worrying, or otherwise distracted. 我没有意识到自己在做什么, 因为我要么在做白日梦、要么在担忧, 不然就在心烦意乱。	0.69				
KIMS 2: I'm good at finding the words to describe my feelings. 我很善于找到描述我感受的词汇。		0.69			
KIMS 26: Even when I'm feeling terribly upset, I can find a way to put it into words. 即使我感到非常心烦意乱时, 我也能找到一种用言语表达它的方式。		0.75			
KIMS 34: My natural tendency is to put my experiences into words. 我会自然地将自己的感受经历用言语表达出来。		0.82			
CAMS 5: I can usually describe how I feel at the moment in considerable detail. 我通常可以详细描述我某一刻的感受。		0.80			
KIMS 12: I tell myself that I shouldn't be feeling the way I'm feeling. 我提醒自己, 不应该用现有的方式去感受外在。					0.57
KIMS 16: I believe some of my thoughts are abnormal or bad and I shouldn't think that way. 我认为自己的有些想法是不正常或错误的, 其实我不应该这样想。					0.73
KIMS 28: I tell myself I shouldn't be thinking the way I'm thinking. 我告诉我自己, 我不应该用现有的方式思考。					0.75
KIMS 32: I think some of my emotions are bad or inappropriate and I shouldn't feel them. 我认为自己有一些不好的或不恰当的情绪, 其实我不应该有这些情绪。					0.72
Cronbach's alpha for each factor (value for whole scale = 0.73)	0.80	0.79	0.75	0.67	0.66

The relationship between FFMQ and SF-FFMQ was also verified. The correlation between the two instruments is 0.70 and significant (*p* < 0.05). The internal consistencies of each of the five facets and the whole FFMQ in Sample 1 were highly variable, as indicated by the following Cronbach's alpha values: non-reactivity, 0.67; observe, 0.75; act with awareness, 0.80; describe, 0.79; non-judging, 0.66; and whole FFMQ, 0.73. The mean scores (SDs) of each facet and the whole scale obtained in Sample 1 were as follows: non-reactivity, 3.14 (0.66); observe, 2.63 (0.78); act with awareness, 3.40 (0.72); describe, 3.05 (0.70); non-judging, 2.97 (0.63); and whole FFMQ, 15.20 (1.76). Mean FFMQ scores did not differ significantly between men [15.35 (1.82), *N* = 226] and women [15.09 (1.71), *N* = 309; *t*_(535)_ = 1.635, *p* = 0.103].

Subsequent maximum-likelihood CFA in Sample 2 to test the aforementioned five-factor structure in Sample 1 were as follows: χ^2^ = 367.824, *df* = 160, χ^2^/*df* = 2.299 (*p* < 0.001); GFI = 0.92; NFI = 0.86; IFI = 0.92; TLI = 0.90; CFI = 0.92; and RMSEA = 0.058. Generally, the goodness of fit indices was acceptable indicating that the five-factor structure of FFMQ was supported. The above results confirmed the five-factor structure of FFMQ with a good model fit. The AVEs of each factor were shown on the diagonal in Tables 2, 3.

**Table 2:** Correlations among variables in Sample *1*. *(N* = *535)*.

**Variables**	**(1)**	**(2)**	**(3)**	**(4)**	**(5)**	**(6)**	**(7)**	**(8)**	**(9)**
(1) Age	1.000								
(2) Gender	0.321^**^	1.000							
(3) Married	0.689^**^	0.190^**^	1.000						
(4) Non-reactivity	0.073	0.065	0.045	(0.48)					
(5) Observe	−0.079	0.018	−0.091^*^	0.134^**^	(0.55)				
(6) Act with awareness	0.143^**^	0.023	0.076	0.187^**^	−0.248^**^	(0.58)			
(7) Describe	0.028	0.042	0.040	0.347^**^	0.159^**^	0.141^**^	(0.59)		
(8) Non-judgment	0.087^*^	0.028	0.092^*^	−0.068	−0.167^**^	0.280^**^	−0.041	(0.47)	
(9) Mindfulness	0.093^*^	0.068	0.056	0.621^**^	0.394^**^	0.524^**^	0.640^**^	0.357^**^	1.000

*N = 535*,

* *Shows significance at the 0.05 level*,

** *shows significance at the 0.01 level. The AVEs of five factors are shown on the diagonal*.

**Table 3:** Correlations among variables in Sample 2 (*N* = 391).

**Variables**	**(1)**	**(2)**	**(3)**	**(4)**	**(5)**	**(6)**	**(7)**	**(8)**	**(9)**	**(10)**	**(11)**	**(12)**	**(13)**	**(14)**	**(15)**	**(16)**	**(17)**	**(18)**	**(19)**	**(20)**
(1) Gender	1.000																			
(2) Age	−0.058	1.000																		
(3) Race	−0.044	−0.043	1.000																	
(4) Education	0.089	−0.233^**^	−0.053	1.000																
(5) Organization type	−0.188^**^	0.464^**^	−0.009	−0.058	1.000															
(6) Work experience	−0.092	0.949^**^	−0.045	−0.326^**^	0.469^*^	1.000														
(7) Nonreactivity	−0.010	0.018	−0.022	−0.005	0.144^**^	0.045	(0.50)													
(8) Observe	−0.086	−0.019	0.048	−0.052	0.160^**^	0.005	0.265^**^	(0.56)												
(9) Act with awareness	0.184^**^	−0.020	−0.027	0.067	−0.221^**^	−0.033	−0.092	−0.373^**^	(0.56)											
(10) Describe	0.041	0.076	−0.062	0.056	0.221^**^	0.068	0.253^**^	0.423^**^	−0.208^**^	(0.63)										
(11) Non-judgment	0.071	−0.014	0.075	−0.043	−0.130^*^	−0.042	−0.274^**^	−0.392^**^	0.382^**^	−0.395^**^	(0.45)									
(12) Mindfulness	0.088	0.020	0.004	0.012	0.102^*^	0.023	0.555^**^	0.535^**^	0.292^**^	0.582^**^	0.067	1.000								
(13) SAS score	−0.160^**^	0.136^**^	−0.025	−0.157^**^	0.106^*^	0.156^**^	0.004	0.198^**^	−0.401^**^	−0.024	−0.235^**^	−0.193^**^	1.000							
(14) CES-D score	−0.143^**^	0.073	−0.017	−0.091	0.104^*^	0.091	−0.060	0.169^**^	−0.385^*^	−0.054	−0.199^**^	−0.230^**^	0.841^**^	1.000						
(15) Life well-being	0.105^*^	0.098	−0.008	0.111^*^	0.069	0.083	0.271^**^	0.089	0.138^**^	0.246^**^	−0.082	0.325^**^	−0.218^**^	−0.318^**^	1.000					
(16) Workplace well-being	0.050	0.012	0.018	0.179^**^	−0.022	−0.000	0.171^**^	0.113^*^	0.124^*^	0.164^**^	−0.164^**^	0.212^**^	−0.155^**^	−0.195^**^	0.642^**^	1.000				
(17) Psychological well-being	0.061	−0.073	−0.008	0.083	−0.045	−0.103^*^	0.355^**^	0.210^**^	0.123^*^	0.272^**^	−0.199^**^	0.385^**^	−0.183^**^	−0.220^**^	0.458^**^	0.524^**^	1.000			
(18) Employee well-being	0.087	0.020	0.001	0.151^**^	0.003	−0.002	0.311^**^	0.160^**^	0.154^**^	0.269^**^	−0.174^**^	0.362^**^	−0.222^**^	−0.293^**^	0.853^**^	0.877^**^	0.768^**^	1.000		
(19) Servant leadership	−0.097	0.049	−0.013	0.083	0.239^**^	0.040	0.317^**^	0.218^**^	−0.079	0.276^**^	−0.271^**^	0.247^**^	0.013	−0.030	0.301^**^	0.444^**^	0.278^**^	0.412^**^	1.000	
(20) Satisfaction with work-family balance	0.084	0.008	0.006	0.091	0.067	−0.019	0.283^**^	0.200^**^	0.010	0.280^**^	−0.197^**^	0.298^**^	−0.108^*^	−0.169^**^	0.527^**^	0.488^**^	0.458^**^	0.589^**^	0.296^**^	1.000

CES-D, Center for Epidemiological Studies Depression Scale; SAS, Self-rating Anxiety Scale;

* *Shows significance at the 0.05 level*,

** *shows significance at the 0.01 level. The AVEs of five factors are shown on the diagonal*.

The correlations among the five facets in two samples are shown in Tables 2, 3. As can be seen, the pattern is generally consistent among the two samples. However, act with awareness was negatively related to observe (*p* < 0.01), and non-judging was also negatively related with non-reactivity (*p* < 0.01), observe (*p* < 0.01), and describe (*p* < 0.01). We explain these results in the discussion part.

We further examined construct predictive validity in Sample 2 by exploring how FFMQ scores are related to CES-D and SAS scores. Cronbach's alphas for internal consistency were 0.70, 0.81, 0.77, 0.84, 0.69, and 0.85 for non-reactivity, observe, act with awareness, describe, non-judging and the whole FFMQ, respectively. The Pearson's correlation coefficients between mindfulness facets and related constructs in Sample 2 are reported in Table 3. The observe, act with awareness, and non-judging facets correlated with *CES-D* and *SAS* scores. Meanwhile, the non-reactivity and describe facets correlated more strongly with well-being outcomes than the other facets.

### Mediating Role of Mindfulness Between Servant Leadership and Satisfaction With Work-Family Balance

Preliminary CFA examining the discriminant validity of our variables and potential common method bias was completed. Our hypothesized three-factor model (χ^2^ = 231.832, *df* = 87, χ^2^/*df* = 2.665 (*p* < 0.001); GFI = 0.92, NFI = 0.92, IFI = 0.95, TLI = 0.93, CFI = 0.95, and RMSEA = 0.065) fit the data better than a parsimonious two-factor in which mindfulness and satisfaction with work-family balance were combined (χ^2^ = 456.341, *df* = 89, χ^2^/*df* = 5.127 (*p* < 0.001); GFI = 0.84, NFI = 0.83, IFI = 0.86, TLI = 0.84, CFI = 0.86, and RMSEA = 0.10) and a one-factor model in which all three variables were combined (χ^2^ = 1178.647, *df* = 90, χ^2^/*df* = 13.069 (*p* < 0.001); GFI = 0.63, NFI = 0.57, IFI = 0.59, TLI = 0.52, CFI = 0.59, and RMSEA = 0.18). These results confirm the distinctiveness of the variables and demonstrate that the common method variance should not be a substantial problem although the data were collected from a single source.

**Figure 1:**
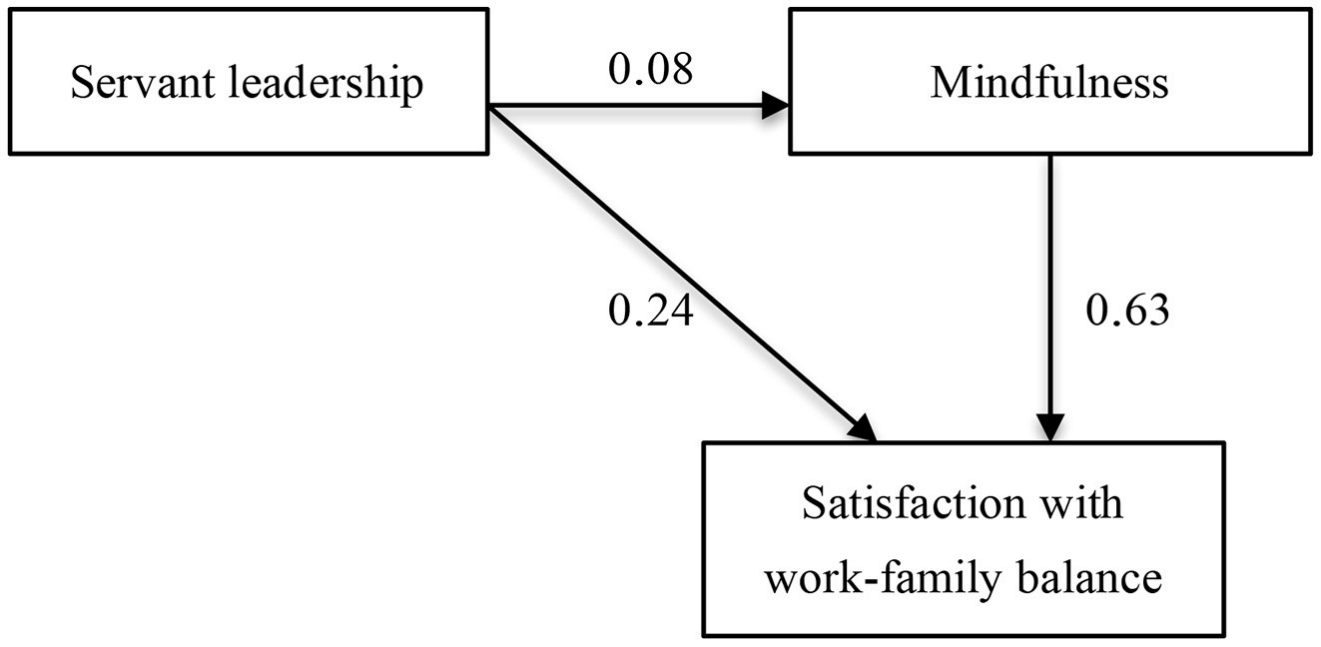
The hypothesized structural equation model with standardized regression weights (all coefficients are significant at *p* ≤ 0.001 level).

Subsequently, we conducted structural equation modeling, with servant leadership and mindfulness as whole constructs, to test the hypothesis that mindfulness skills mediate a positive relationship between servant leadership and follower well-being outcomes in Sample 2. As summarized in Figure 1, our partial mediation model achieved significant results (χ^2^ = 231.832, *df* = 87, χ^2^/*df* = 2.665; *p* < 0.001). Note that we obtained significant paths (all *p* < 0.001) from servant leadership to mindfulness (β = 0.08, standard error = 0.02, 95% confidence interval = 0.05–0.12), from servant leadership to satisfaction with work-family balance (β = 0.24, standard error = 0.05, 95% confidence interval = 0.13–0.34), and from mindfulness to satisfaction with work-family balance (β = 0.63, standard error = 0.14, 95% confidence interval = 0.34–0.89). Confirmatory bootstrapping analysis affirmed a significant indirect effect of servant leadership on satisfaction with work-family balance via mindfulness (*B* = 0.05, standard error = 0.02, *p* < 0.001, 95% confidence interval = 0.03–0.09). Because the 95% confidence interval of the pathways did not include zero, these results support the notion that mindfulness skills of employees mediated a positive relationship between servant leadership behaviors and employee satisfaction with work-family balance.

## Discussion

The present examination of the psychometric properties and factorial structure of the Chinese SF-FFMQ, in Aim 1, yielded acceptable internal consistencies of the instrument as a whole and of its five facets. Both EFA and CFA results supported a five-factor structure of our Chinese SF-FFMQ in two Chinese samples. Importantly, we validated this structure in working adults (as opposed to only in a student sample), helping to expand the scope of the FFMQ's utility. Moreover, mindfulness facets correlated with related constructs, including the CES-D, SAS, and three employee well-being outcomes. With respect to our second aim investigating the potential mediating role of mindfulness in the relationship between servant leadership and satisfaction with work-family balance, we demonstrated that servant leadership related positively to employees' mindfulness skills and influenced employees' satisfaction with work-family balance through the mediating factor of mindfulness.

Our correlation results between mindfulness facets and related variables were largely similar to prior findings by Baer et al. (2006, 2008), wherein four of the five facets correlated with other constructs and three of the five facets were found to be predictive of psychological symptoms. Here, we found that three of the five facets were significantly related to depression and anxiety symptoms. Notably, like Baer et al. (2006, 2008), we found that the observe facet did not fit the CFA model and that it had unexpected relationships with other variables. Although the reasons for these results are not clear, they may reflect the changing nature of this facet and/or sample choice (Gu et al., 2016). Baer et al. (2006) also noted that FFMQ may be best suited for clinical samples. According, our use of non-clinical samples without meditation training may help to explain why the non-reactivity and describe facets were not found to have significant relationships with CES-D and SAS scores.

We found that there are negative correlations among some of the mindfulness facets such as the correlation between observe and act with awareness and the correlation between observe and non-judging in the current study. On one hand, the use of non-clinical sample could be one reason. Earlier studies that reported the positive relations used the clinical sample either with meditation training experiences or psychology study experiences (e.g., Baer et al., 2006). However, the sample of manufacturing industry employees in the current study should have very limited understandings and experiences of mindfulness. On the other hand, such negative correlations between some mindfulness facets were also found in eastern settings. For example, in their development and validation of a Japanese version of FFMQ, Sugiura et al. (2012) found that observe is negatively correlated with both act with awareness (−0.12, *p* < 0.01) and non-judging (−0.32, *p* < 0.01). The reason might be that the eastern sample emphasizes less on their “active attitudes toward experiences” (Sugiura et al., 2012) which refers to observing and describing, but they would focus more on their views and judgments. Therefore, similar pattern of negative correlations among certain mindfulness facets appears in eastern samples. Future research could examine how would the correlations among mindfulness facets differ across different cultural environments.

The five FFMQ facets were mostly positively related to life well-being, workplace well-being, psychological well-being, and whole employee well-being, except that the *observe* and *non-judging* facets were not positively related to life well-being. Indeed, *non-judging* exhibited a negative relationship with employee well-being. Given that each person's opinion about whether he or she experiences well-being is based on his or her own judgment (Zheng et al., 2015), the *non-judging* facet may not necessary relate positively to well-being outcomes, particularly if it relates negatively to dysfunctional attitudes and psychological symptoms. More research is needed to further explore the relationships between the *non-judging* facet and a positive attitude.

The findings of our study aide in integrating mindfulness research with leadership literature. We hypothesized that servant leadership behaviors (i.e., listening, emotional healing, persuasive mapping, and altruistic calling) would lead employees to experience mindfulness changes that may improve the skills of observing, acting with awareness, describing, refraining from judgment, and non-reactivity. The holistic view advocated by servant leaders (Greenleaf, 1977; Spears, 2004) suggests that mindfulness should enable employees to improve work-family balance as a consequence to being mindful at work and at home.

The presently supported mediation role of mindfulness provides evidence that mindfulness is a useful principle for organization science and management research. If mindfulness can be established as a root construct in management studies, then it can help improve our fundamental understanding of how individuals think, perceive, and behave in the workplace and thus advance the impacts of servant leadership. Servant leaders hold a person-oriented attitude toward others, as opposed to focusing on organizational outcomes, and servant leadership has been reported to affect organizations positively in terms of job performance, organizational citizenship behavior, creativity, organizational commitment, team performance, and work engagement (Walumbwa et al., 2010; Hunter et al., 2013; Liden et al., 2014; van Dierendonck et al., 2014; Chen et al., 2015; Chiniara and Bentein, 2016; Neubert et al., 2016). However, the mechanisms by which servant leadership impacts individual outcomes remain to be clarified. It is reasonable to suppose that servant leadership-engendered improvements in problem-solving and easing of pain, anxiety, and sadness would enable followers to attain a higher satisfaction with work-family balance. Mindfulness could be one important mechanism of such effects by way of its influences on attention, cognition, emotion, behavior, and physiology (Good et al., 2016; Creswell, 2017). We are encouraged that future research connecting mindfulness to leadership theories may yield additional fruitful findings.

The present study had some limitations that need to be acknowledged and addressed in future research. First, we employed non-clinical samples without serious dysfunctional attitudes or psychological symptoms. Given that Baer et al. (2006) suggested that the FFMQ was best-suited for populations with mental health problems, future research should examine the factorial structure of the Chinese SF-FFMQ in clinical samples. Such clinical sample might show even better psychometric properties such as internal consistencies and correlations among certain mindfulness facets. Notwithstanding, our data do validate the SF-FFMQ in a working-adult sample, a population that was in need of validation (Baer et al., 2006). Second, because the present data are cross-sectional in nature, we should be cautious regarding inference of causal relationships. Potential causal links should be examined in longitudinal studies. And such longitudinal design could also better examine the relationship between servant leadership and mindfulness. Third, the mediating model we proposed did not consider boundary conditions. For example, some individual differences or contextual factors might moderate our mediation model. Because organizational policies affect employees' work-family balance, future modeling research should examine whether main effects or mediation effects would be impacted by organizational-level factors, such as family support arrangements. Fourth, in order to better demonstrate mindfulness as a root construct in management studies, future study could choose more variables closely related to mindfulness as the calibration standard and then further test the criterion related validity of SF-FFMQ and these variables.

In summary, the present study made several important contributions. First, we provided the first validation of a Chinese SF-FFMQ with acceptable psychometric properties in two Chinese samples indicating that it can be used to assess the five facets of mindfulness. The abbreviated length of this instrument reduces participant burden. Second, we found that this Chinese SF-FFMQ predicted some psychological symptoms, dysfunctional attitudes, and well-being outcomes. Importantly, we showed that it could be utilized in working adult samples. Third, this study is the first to link servant leadership theory with mindfulness, thereby advancing our understanding of mindfulness' unique role in management studies and contributing to servant leadership knowledge. The present findings are consistent with the possibility that mindfulness could be developed into a root construct of organization science.

## Data Availability Statement

The datasets generated for this study are available on request to the corresponding author.

## Ethics Statement

The studies involving human participants were reviewed and approved by Ethical committee of Xinxiang Medical School, Ref No. 2017-00031. The patients/participants provided their written informed consent to participate in this study.

## Author Contributions

YM and KM: data collection, conception and design, data analysis, and writing the paper. CL: research design and critical revision.

### Conflict of Interest

The authors declare that the research was conducted in the absence of any commercial or financial relationships that could be construed as a potential conflict of interest.
